# Understanding and supporting law enforcement professionals working with distressing material: Findings from a qualitative study

**DOI:** 10.1371/journal.pone.0242808

**Published:** 2020-11-25

**Authors:** Cristina-Bianca Denk-Florea, Benjamin Gancz, Amalia Gomoiu, Martin Ingram, Reuben Moreton, Frank Pollick

**Affiliations:** 1 Institute of Neuroscience and Psychology, The University of Glasgow, Glasgow, United Kingdom; 2 Qumodo, London, United Kingdom; 3 Department of Psychology, The Open University, Milton Keynes, United Kingdom; TNO, NETHERLANDS

## Abstract

This study aimed to extend previous research on the experiences and factors that impact law enforcement personnel when working with distressing materials such as child sexual abuse content. A sample of 22 law enforcement personnel working within one law enforcement organisation in England, United Kingdom participated in anonymous semi-structured interviews. Results were explored thematically and organised in the following headings: “Responses to the material”, “Impact of working with distressing evidence”, “Personal coping strategies” and “Risks and mitigating factors”. Law enforcement professionals experienced heightened affective responses to personally relevant material, depictions of violence, victims’ displays of emotions, norm violations and to various mediums. These responses dampened over time due to desensitisation. The stress experienced from exposure to the material sometimes led to psychological symptoms associated with Secondary Traumatic Stress. Job satisfaction, self-care activities, the coping strategies used when viewing evidence, detachment from work outside working hours, social support and reducing exposure to the material were found to mediate law enforcement professionals’ resilience. Exposure to distressing material and the risks associated with this exposure were also influenced by specific organisational procedures implemented as a function of the funding available and workload. Recommendations for individual and organisational practices to foster resilience emerged from this research. These recommendations are relevant to all organisations where employees are required to view distressing content.

## Introduction

The introduction of landmark technologies such as the Internet and smartphones led to the development of cybercrime (e.g. hacking and malware attacks) and opened new avenues for traditional offences where the computer can be used as a tool to commit crimes. Examples of traditional crimes that have expanded into the digital sphere include terrorism, fraud and paedophilia [1, 2]. The emergence of cybercrime has also led to a paradigm shift in criminal investigations. Historically, the key police investigative aids were eyewitness testimony and physical forensic evidence, yet they are no longer relevant in cyber contexts. In cybercrime, digital evidence of the crime can be enough for prosecution and conviction.

Looking at the influence of cybercrime on Child Sexual Abuse Material (CSAM), we observe that CSAM can be found on multiple online platforms, from image hosting sites and third-party online services which provide file-storing and file-sharing services to commercial web streaming and social networking sites [3]. Looking further into the volume of the material encountered, the Internet Watch Foundation [4] has identified 132,676 websites in 2019 alone containing CSAM, with new websites being constantly discovered. The severity of the problem is also observed when looking at the statistics provided by the National Crime Agency of the United Kingdom (UK) showing that, in 2018, 2.88 million accounts were registered globally across the most harmful CSAM dark websites [5]. This occurred despite the fact that the production, access, possession and distribution of this material is illegal in many countries. Apart from CSAM, other types of illegal material that can be found online depict paraphilias and highly violent content [6–10].

In the course of investigations, law enforcement professionals (LEP) may deal with extremely large amounts of evidence containing millions of images and videos spread across multiple devices [11]. While not all content they view is illegal, it is estimated 70–80% of digital forensic investigation cases involve CSAM [12]. Apart from CSAM, members of law enforcement (LE) also view other types of highly violent material such as bestiality, snuff films [9] etc. Exposure to this type of material occurs during various tasks, such as when checking websites for illegal content, classifying the material according to the content depicted, preparing reports detailing selected images and viewing those selected images in the course of prosecution [13]. Exposure to illegal material such as CSAM is fundamentally distressing, and some LEP suggest that this is the most difficult aspect of their work [8, 10]. Indications of the material’s unpleasantness are also found within the physical and emotional responses they elicit. Typical descriptors of reactions include nausea, sadness, anger, frustration and shock [9]. Looking at the specific features driving the emotional response, content depicting violence, displays of emotions from the victims, norm violations, the personal relevance of the material to the viewer, and the medium of the material were all reported to influence LEP’s affective reaction [6–10, 14].

Aside from the difficulty in viewing distressing material, this exposure may have a severe impact on LEP’s long-term welfare. This is because contact with other people’s traumatic experiences can lead to the development of trauma-associated symptoms. While this is referred to as Secondary Traumatic Stress (STS) in the literature [15], recently it was formally recognized as a form of Posttraumatic Stress Disorder (PTSD) [16]. These symptoms were initially documented in professions where people are often exposed to human suffering and death, either through narrations of distressing events, as in the case of mental health staff [17–20], or through direct exposure to those events, as in the case of police professionals [21–24], medical personnel [24–26], firefighters [24] etc. More recently, STS symptoms were also reported by LEP as a result of exposure to traumatizing events through digital media files [6–10, 14, 27–30], indicating the effects go beyond direct contact with traumatized victims. In LEP exposed to digital distressing material, STS symptoms are manifested in the form of intrusive thoughts, avoidance, and alterations in mood, arousal and reactivity [6–10, 14, 27, 28].

While the negative impact of viewing distressing material is clearly documented among LEP, members of LE are not equally affected. This was illustrated by multiple studies which found variations in the severity of STS symptoms among LEP working within the same agency [8, 27, 28]. Differences in the severity of impact were also found across agencies located in different countries. For example, when comparing the STS scores of a UK sample of LEP with a sample of LEP from the United States of America (USA), Bourke and Craun [28] found that 10.4% of British LEP scored in the severe category of STS as compared to 15.3% of USA personnel. Also, a larger proportion of British LEP scored in the low or no STS category (36.9%) as compared to its USA counterparts (26.4%). These differences in impact can be explained by personal factors such as one’s social support net both inside and outside of work [6], one’s level of engagement in coping strategies and the specific type of coping strategies used [28]. However, personal factors are not the only contributor to LEP’s resilience to the job, their welfare also being dependent on their agency’s practices. Aspects related to recruitment, supervision, workload, training, equipment, procedures related to viewing evidence and access to professional support are all considered by LEP to play a role in their well-being [6, 14, 31, 32]. For example, excessive workload [6, 14, 32], poor supervisory support [32] and a lack of training in self-care and the technical aspects of their jobs [14] are all viewed by LEP to hinder their well-being.

In light of the previously mentioned mental health risks associated with LEP’s need to view distressing material as part of investigations and the ever-increasing discovery of such content [3, 4], the safeguarding of LEP’s mental health must take precedence. Considering the observed variations in impact and coping among LEP within and between different agencies [8, 27, 28], more research is needed to improve our knowledge of LEP’s experiences. In doing so, we aim to build a more comprehensive picture of how viewing distressing material affects LEP and how these effects may be mitigated. The current research seeks to provide further understanding of UK LEP’s direct experiences with viewing distressing material and the corresponding factors modulating their well-being. We chose to investigate these aspects using qualitative methods in order to be able to capture the inherent complexity of factors associated with LEP’s experiences [14]. Also, qualitative methods may be better at discovering stressors and/or coping strategies not identified through other forms of investigation [33].

## Methods

### Participants

Twenty-two law enforcement personnel (16 males, mean age = 40.65, SD = 11.91, age range = 26–67) working in a Police Force in England, UK participated in the study. Through purposive sampling, the participants were recruited with the help of managerial staff who sent out an email with the advertisement of the study within the organisation. The email set out a brief summary of the aims of the study and an outline of how the study would be undertaken. Following this email, recruitment also occurred through snowballing. The participants interested in taking part in the study contacted the researcher via the email provided in the advertisement. We only included participants who were exposed to distressing material in their current and/or previous roles. Recruitment was stopped when recurrent patterns became evident in the participants’ narrations, indicating data saturation was reached. Our final sample mostly consisted of digital forensics technicians (59%), while individuals with the roles of manager, investigator, supervisor and detective were also identified (see S1 Table for more detailed participant characteristics). Tenure of employment in their current roles ranged from 1–17 years (M = 4.72, SD = 4.28). All participants provided written informed consent prior to the beginning of the study and were reimbursed for their participation at the rate of £6/hour. Participants were assured of anonymity and were only identified by a numerical code. Ethical approval for this study was obtained from the College of Science & Engineering at the University of Glasgow under the application number 300180129.

### Procedure

#### Research team and reflexivity

The principal investigator, CBDF, conducted the interviews in June 2019, in private rooms located in the participants’ workplace. There was no relationship between the research team and the participants prior to commencing the study, and the participants were told that the interviewer was a university researcher.

#### Study design

A semi-structured, one-to-one interview schedule was developed based on a review of the literature in the area of law enforcement. The interviews lasted between 8 and 50 minutes (Mean interview duration = 22.36, SD = 10.10) and were conducted face to face by the principal investigator. The interview questions were open-ended in order to encourage elaboration, seek clarification and to allow the participants to raise and discuss issues that were important or interesting to them. The questions (see S2 Table) mainly focused on the participants’ perceptions and experiences of working with distressing imagery. This included questions about the participants’ subjective experience viewing distressing imagery, their emotional reactions to the imagery and their evaluation of factors that hinder or support their welfare in the context of their role. Participants also optionally provided basic demographic information (gender, age, ethnicity, marital status and education level).

#### Analysis

All interviews were audio-recorded and transcribed verbatim. Data was manually transcribed by CDF using InqScribe 2.2.4 [34]. Transcripts were double-checked for accuracy against the original recordings. Data were analysed using inductive thematic analysis at semantic level [35]. We chose this analysis as it allowed us to give voice to the participants’ experiences and meanings attached to them as reported in the data. Furthermore, this analysis gave us the opportunity to consider the social and psychological interpretations of the data.

Following a process of data familiarization (i.e. reading and re-reading of the data) and in order to improve the reliability of the analysis, the transcripts were independently coded by three researchers with previous experience in qualitative data analysis (CBDF, AG, MI). The researchers carried out the analysis following the thematic analysis guidelines provided by Braun and Clarke [35]. Coded data were independently sorted into meaningful themes and all data relating to each theme were collected. The researchers then met and mapped, revised and refined the themes and subthemes to ensure a good fit with the data. Emerging patterns were also considered in the light of empirical literature, which helped to set the analysis of this study within the context of broader research. In order to check that the themes reflected the experience of the participants in a believable way, the extracted themes were refined after validating them with selected participants [36]. Grammatical changes were made to the quotations supporting the themes in order to improve flow and clarity. Any detail that could potentially lead to the identification of individual participants, victims or suspects was removed or redacted.

## Results

The final thematic map identified 4 overarching themes (Table 1) of specific relevance to this study as set out below together with their subthemes.

**Table 1:** The emerging themes and subthemes of the analysis.

Themes	Subthemes	Number of Participants (Percentage of total)
Reactions to the evidence	Immediate responses	12 (55%)
	Desensitisation	12 (55%)
	Evidence features influencing reactions	16 (72%)
	Personal relevance	10 (45%)
	Medium	10 (45%)
Impact of working with distressing evidence	Intrusive images and thoughts	19 (86%)
	Protectiveness and mild paranoia	5 (23%)
Personal coping strategies	Viewing strategies	16 (73%)
	Talking	15 (68%)
	Humour	7 (32%)
	Relaxing activities	9 (41%)
Risks and mitigating factors	Supervision	11 (50%)
	Psychological support	12 (55%)
	Physical workspace	11 (50%)
	Volume & Frequency of exposure	22 (100%)
	Rationale for the job	16 (73%)
	Social support	8 (36%)
	Resilience	11 (50%)

### Responses to the material

The participants talked about their immediate and long-term responses to the material and about the individual factors that influenced their engagement with it. These are given below.

#### Immediate responses

In current and previous roles, the participants were exposed to a wide range of material falling under both the legal and illegal spheres. With respect to the material depicting illegal practices, the themes frequently mentioned included CSAM, extreme violence and torture of humans and animals, extreme pornography (e.g. rape, bestiality) and snuff videos (e.g. beheadings, suicides). Nine of the participants reported a mix of responses associated with this material, from emotional responses of shock, anger, and empathy for the victim to feelings of inadequacy and physical responses of sickness. Three participants did not name any specific emotions that they experienced. However, two of those participants described having to occasionally look away from the material or having the desire not to look at the material, indicating they also had a negative response to the content.

*“I think maybe shock if anything else because you see things that you would not normally see*. *Yes*, *shock and disgust that there are people like that around and it can be anyone and obviously feeling sorry for the victims as well*.” *(Digital forensics technician)*

#### Desensitisation

Participants mentioned changes in their affective responses to viewing distressing material. When participants first viewed the material, they experienced distress, yet over time they gradually became desensitised to it. This came as a result of repeated exposure, leading to a reduction in the strength and amount of negative emotions experienced. After this point, the participants only reported an amplified negative response to novel content. Desensitisation seemed to occur for any type of distressing imagery the participants encountered. Two participants felt uncomfortable with this change in response and questioned whether this dampening of affect was personally beneficial. These concerns were discussed in relation to possible alterations in the participants’ core principles of what is right and wrong behaviour.

*“When I first started here*, *I had a strong adverse response*. *You start to look at these things and then you feel physically sick*, *a bit lightheaded because they are quite disturbing*, *but it is amazing how fast you adapt*. *It is repetition*. *Each time you feel a bit better enough to after a few weeks of doing it is like almost nothing really*. *You do not know how it affects you subconsciously*, *but it does not appear to*, *no*. *I would happily eat sandwiches while doing it*.” *(Investigator)*

*“The more you see you become a bit desensitised to it*, *it becomes a bit nonchalant and blasé and that is maybe not where you should be*. *You should always be mindful of what you are doing*, *I think*.*” (Digital forensics technician)*

#### Features influencing reactions

When viewing case materials, participants reported having expectations about the type of content they expected to affect them. However, these expectations were not often met, and participants realised that they cannot always predict the type of material that will trigger a response. Among the content perceived to be inducing an emotional response, fourteen participants found content which violates norms to be highly disturbing. Participants reported being particularly distressed by norm violations within CSAM, especially when viewing female perpetrators, first generation material, and when the offence was committed by a known family member. Furthermore, the material was viewed as most disturbing when the victims were very young (i.e. babies and toddlers) and when there was a big age difference between the offender and the victim. A combination between the age of the victim, sexual abuse and physical abuse was perceived to heighten distress even more. Other content considered to be particularly distressing contained depictions of homicide and highly violent and sadistic behaviour. The determination of the perpetrator and the total duration of the abuse were other features that induced distress further.

*“I think there is definitely something about kids and one’s natural desire to protect tiny cute things which is the only reason that parents are not too angry at their annoying children because they are small and cute*. *There is definitely something about that that makes it for most people*, *probably myself included*, *often more distressing than some other harm*, *but it also depends what you are used to seeing*.*” (Digital forensics technician)*

*“Full grown adults and toddler babies and they were just abusing them*. *It was really quite hard and quite disturbing*. *They should not be doing that*, *should they*?*” (Digital forensics technician)*

The victims’ behaviour was mentioned by five participants as a contributor to their emotional responses of distress. Overt negative emotional responses of the victims such as crying were described as upsetting. These displays of emotions were perceived to be especially distressing when children were the victims. Within the context of CSAM, the lack of any overt indications of distress from victims, or contrarily, displays of positive emotions from the victims were interpreted as signs of sustained grooming endured by the victim, or the victim’s confusion or desensitisation to the abuse.

*“Knowing that someone close is doing the abuse is more affective*. *That really upsets me*. *Especially when you see they have taken pictures of what they have done*. *They put the person in certain positions and sometimes you see the subject’s face and you can see they are distressed*. *It is very upsetting*.*” (Digital forensics technician)*

*“How the victims acted like it was normal*. *When they were abused it was like a game or a bit like a joke*. *[…] It just makes you think this has been going on for a long time because you look at different reactions from the victims and then who do you feel sorrier for*? *It might even be a child who is 12 and they seem to be enjoying it*, *but you know that if you look at it further that is not the case at all because that is something that has been going on since they were maybe 5 or they do not know any better or any different*. *So*, *I think it is one of the things I have found very difficult to see*.*” (Digital forensics technician)*

#### Personal relevance

The relevance of the material to the participants’ personal lives was another factor that could influence their reactions. For example, participants who had children of their own have reported greater difficulty viewing images of child abuse. Similarly, establishing rapport with the victim or noticing parallels between their own lives and the victims’ life were also mentioned as especially distressing by our participants. This was because instances like these have enhanced the viewers’ ability to relate to and create a connection with the victims.

*“It did not affect me that much when I first started in digital forensics*. *Since then it has increased but I was off for a while*. *Coming back and doing it*, *it has affected me a lot more because I now have children and I guess you put yourself in their shoes and it hits home more in some way*. *(Digital forensics technician)*

*“The only time I would say I ever felt affected was not even to do with any indecent images*. *It was to do with suicide where this person has committed suicide*. *They jumped in front of a train and they were my sibling’s age*. *I remember thinking a lot about that on my way home*.” *(Digital forensics technician)*

*“If someone looks like a child you know that can then be quite difficult to deal with or it hits you that little bit more*.” *(Digital forensics technician)*

#### Medium

The strength of the emotions experienced was also dictated by the medium of the material. Video material was perceived to be highly distressing as it forced the participants to view the offence as if it were taking place again, and thus witness the trauma experienced by the victims. Multisensory content such as video combined with audio and written information were also reported to be highly distressing because they enhanced the participants’ ability to recollect the events. Adding to the vividness was the quality of the material, high-quality imagery being perceived to be more impactful.

*“The videos have more of an impact because it is more of a story*. *If you just have a single image*, *then you tend to make up the story in your head whereas when you have something physically there and you can watch it from beginning to the end the story tells itself*.” *(Digital forensics technician)*

*“One of the first times I experienced a nightmare*, *[…] it was a written description of something*. *It was in relation to a murder of a child who was linked to someone who I was investigating and weirdly that is probably the worst because I think it makes you create the image in your head rather than see it on a screen*. *We are so used to desensitising ourselves to images we see on a screen so that is certainly the worst*.” *(Detective)*

### Impact of working with distressing evidence

Participants described multiple effects following exposure to distressing material, which manifested as intrusive images, unwanted thoughts, and mild paranoia. These effects were not universal, two participants reported not being affected by viewing the material. While it is possible that the participants did not suffer any impact of exposure, it is also possible they were not aware of the impact. This was suggested by three participants who mentioned having difficulty in identifying if their exposure to this material affected their own well-being. However, one of these participants mentioned that while they did not notice the effects when engrossed in that work, these became apparent once their exposure stopped.

#### Intrusive images and thoughts

Seven participants reported experiencing flashbacks, which they related to viewing distressing material. These were triggered by environmental aspects they associated with the events depicted in the material. Most notably, flashbacks occurred for some participants when they interacted with children they knew personally outside of their work environment. Likewise, some participants also described flashbacks occurring during intimate moments between the participants and their partners. Adding to these flashbacks were somewhat arbitrary and case-specific features, which some participants associated with the explicit materials. These could be anything from auditory to visual or written information. Some examples included hearing a song associated with sexual abuse, seeing a certain colour worn by a victim, or hearing a person use wordings that were associated with terminology used by the perpetrators. These effects often impacted on the participants’ behaviours, such as avoiding specific activities in order to reduce the incidence of these flashbacks. Seven participants also mentioned thinking about the material outside of work, with some experiencing nightmares. Generally, these events were reported to be short lasting, occur only sporadically, and seemed to have limited impact on the participants’ lives and their ability to work in this area. Also, participants noted that some of these effects tended to disappear once they acquired more experience of the job.

*“I viewed certain videos during the week as part of the investigation I was helping with and then I went at the weekend to see my friend who has a child and we were looking after him and they changed his nappy and I just had a sudden flashback to the video that I saw earlier in the week*. *I felt I could not go near him because it was going to trigger back this reaction of this video that I have seen in the week*.*” (Digital forensics technician)*

*“When I was doing baby changes and similar activities… those were the things where ‘Oh*, *wow*, *a memory came into my mind*!*’*. *So*, *take a moment*. *Bath time as well… It could be quite difficult looking at bath time*. *It is just thinking about those people who abuse children*: *‘Wow*, *here that person took advantage of that situation that I’m in now*. *Why would someone do something like that*?” *(Digital forensics technician)*

*“There is still one video… I think it is not as bad as it used to be but there was one video where there was a child in a bathtub being abused and she had on a yellow dress and whenever I saw that shade of yellow*. *So*, *I would be in a completely normal situation where…I remember being shopping and there was a girl with a yellow bag and straight away that video came playing in my head*.” *(Manager)*

*“You can wake up with nightmares from them but not often otherwise you would not be able to do the job and that is the overwhelming exception rather than the rule*.” *(Detective)*

#### Protectiveness and mild paranoia

For some participants, this exposure to distressing materials made them become more cynical about the world. This change manifested in diverse ways such as mild paranoia, overprotectiveness of children, and greater distrust of others. To exemplify, one participant disclosed being paranoid about how their behaviour with children will be interpreted by other people, yet at the same time admitted constantly analysing the interactions between other adults and children. Overprotectiveness manifested itself as setting limits in the activities their children or children of their relatives engaged in due to their professional experiences, and a heightened sensitivity to any potential dangers.

*“I think what it did is it affected my view of the world and people*. *Just knowing that there are people like that in the world*, *who are capable of doing these things*. *I think you cannot fully understand that unless you see it*. *[…] But I think it was not just that*, *it was just doing the work in general and having that insight into people’s lives*. *So*, *it made me a bit more bitter about people*.” *(Digital forensics technician)*

*“We go to social events as family groups together and there will always be children around us and in every normal world there is nothing wrong with a child coming up to you who knows you because you are a trusted part of this group and saying to you ‘Can you take me to do this or that*?’. *I know their parents very well*, *they know me very well*, *but I would question myself quite a lot*: *‘What if somebody sees me taking this child to get some ice cream*? *What would they think of me*?*’*. *I question not my intention because my intention I know of*, *I am not doing anything wrong*, *but I feel like I am putting myself at risk because I am seeing everything from this tinted glass*.*” (Manager)*

### Personal coping strategies

Participants described several personal strategies that helped them cope, which they engaged in both at work and outside of work. These include strategies used when examining evidence, social interactions and activities aimed at reducing stress.

#### Viewing strategies

Some participants started engaging in coping strategies even before they were exposed to explicit materials. This took the form of mentally preparing themselves for viewing the material. During the analysis of the material the participants actively engaged in techniques to limit their exposure to these materials by using the technological tools available. Flipping images to another view, looking at them in thumbnail form rather than full screen, watching videos without the sound on, or only watching short snippets of videos were some of the mentioned strategies. While these strategies were helpful in reducing the amount of exposure to the material, there were also times when the participants had to examine the evidence in more prolonged detail. In these situations, the participants reinterpreted how they perceived the material by either pretending that the events depicted were not real or they engaged in a highly analytical perspective, wherein they focussed more on convicting the perpetrator(s). Four of the participants also mentioned shutting down their emotions when viewing the material. Lastly, one participant also mentioned coping by trying to understand the perpetrators’ rationale behind their behaviour.

*“The way I deal with it is I see it as almost…you know how you can watch a film and it is not real life*. *I do not think of that*, *as that is someone*, *I think of that as that is the child that exists if that makes sense*. *It is literally like a movie*.” *(Digital forensics technician)*

*“I go and switch off*. *I do not think*. *Someone might think that is not the healthiest way of dealing with it*, *but I can*, *to a degree*, *detach myself emotionally because I understand what we are doing needs to be done*.” *(Digital forensics technician)*

*“Understanding why somebody feels a certain way towards a person of a certain age will help you understand the problem more*, *which I think then can link into if you are looking at something and think ‘That is terrible*!*’ versus ‘Why do they find this certain picture or this video attractive*? *Why have they downloaded it*?*’*. *[*…*] Just trying to understand the problem really*.” *(Digital forensics technician)*

Despite using these coping strategies, participants admitted there were times when they felt emotional when viewing these materials. On these occasions, taking a break was found to be most helpful. During these breaks the participants would actively remove themselves from their immediate working environment, by stepping away from their desk and engage in relaxing activities such as going for a walk, having a warm beverage or talking to their colleagues.

#### Talking

Participants feel they benefit from receiving support from their colleagues. This support manifested itself in the form of talking about their feelings towards the material, which allowed them to release built-up tension. When participants talked about their experiences, this openness was limited to their colleagues with whom they had shared experiences. In contrast, the participants found themselves unable to discuss their experiences with people close to them outside of work, such as family and friends. This was because the participants were often concerned that revealing the content of the material that they had seen would traumatize others or create concern around themselves. Only one participant disclosed sharing some of their experiences with people close to them outside of their work environment and this was because these people had similar knowledge and skills that helped them understand their experiences.

*“I expressed it to my colleagues [difficulty dealing with the images]*. *That is the beauty of working in the same environment because you can have stories with one another and then talking and having similar experiences with one another that can help those common situations*.” *(Digital forensics technician)*

*“I do not talk to my partner about it*. *I may go home to my partner and say*: *‘I saw some horrible stuff today so I might not be in the best mood in bed or we are definitely not having sex tonight because today is not the night that that is ok’ but I will not go into detail because I am not sure that is necessary*. *They did not sign up to do this job and I do not think that is a fair emotional burden to put on a person*.*” (Digital forensics technician)*

#### Humour

Participants often used humour to release tension and to bond with their colleagues, which could take the form of both light-hearted and dark humour. Dark humour in particular was perceived to only be appropriate within their occupational environment, and outsiders were not being expected to understand it.

*“There is a lot of black humour people use to deal with these things which for people who are not experiencing the situation will probably find it strange*, *but people laugh and joke about these because that is a way of dealing with it and almost normalizing it and making it seem less serious than it is*.” *(Supervisor)*

*“I think quite a lot of people deal with this stuff by making jokes just to deal with what they are seeing*. *When I first got into the industry*, *I overheard a joke somebody told which I was quite offended by as I was quite new to the industry but over time you understand it is a coping mechanism some people use*.” *(Digital forensics technician)*

#### Relaxing activities

Participants described several activities they engaged in outside of work, which they believed could indirectly improve their ability to cope. Examples of these activities included regular intense exercise, grounding activities such as yoga, taking walks and meditating, as well as watching TV, reading, petting animals and gardening. Overall, these activities helped them relieve stress, switch off and generally relax.

*“I go running every day or go to the gym every day*, *meditate*, *do yoga*, *anything which I can use to switch my brain off*. *If I am doing something where my brain has become more unconscious*, *if you are running*, *meditating*, *it gives you an opportunity for thoughts to flow in your mind rather than fixating on them*.*” (Supervisor)*

*“I love working in my garden*. *There is always work to do in the garden*, *so I always take myself off in the garden where I have my music or reading my papers*.” *(Investigator)*

*“The gym I think is the most effective I think at getting a lot of the stress out and just focusing on training at the gym*. *That is probably the most effective*.” *(Digital Forensics Technician)*

### Risks and mitigating factors

Many factors could either help participants to better cope with their work, or by contrast they could make things more difficult for them. Most of these revolved around organisational support in the form of supervision, psychological support, their physical workspace, the volume and frequency of exposure to these materials, as well as discussions about resilience, social support and appropriate sentencing.

#### Supervision

Participants found that receiving appropriate support from their managers added to their sense of well-being. The managers that were viewed as supportive promoted a relaxed work environment, were approachable, and understood the impact and nature of the job, whilst providing reassurance when needed.

*“Management is always there*, *and they are very*, *very supportive and if you have a question or a problem you go to them and talk about it*. *A lot of the time when we work on jobs like that […] our management would say ‘Oh*, *this is the second job you have done like that this month*, *maybe someone else should do it*.*’ So*, *there is a bit of compassion which is really important*, *I think*.” *(Digital forensics technician)*

*“I remember my boss at the time did something useful when he went through and said this is normal behaviour*. *Looking at something and feeling–whether you are feeling angry towards it—that is normal behaviour to seeing that and I thought that was a really good way of teaching us what is right and wrong*.*” (Manager)*

Conversely, management lacking in these skills, possibly due to inexperience with the job, appeared to negatively affect participants’ well-being. This was extensively illustrated by one case where the participant unsuccessfully requested help from management after struggling when viewing explicit materials, and ultimately went on to develop feelings of anxiety and depression as a result of having to continuously prove their distress. Along the same lines, two participants speculated that some employees may not request help when they are struggling, due to the stigma associated with admitting to this.

*“We used to have managers who had done the work and understood the nature of it*. *Now we do not*. *The support we get has been dramatically diminished and there is much more an attitude of ‘Well just get on with it’*.*” (Detective)*

*“I spoke to management about it [not being able to cope]*. *They did not really believe me that I was having a problem and so asked me to prove it*, *go to the doctors*. *At the moment there has been an attempt to not let me be exposed to those images anymore which I am fine not to do*, *I know I am ok not to see those images but at the same time I still want to be a team player*. *They [management] just said ‘It would just be easier if we take you off those kind of cases’ and it has ultimately led to me moving to another unit where they do a lot less of that stuff but I have assured them as well*, *those people as well*, *that*, *if these images come up*, *I am not going to freak out*, *I have still worked with them in the past year*. *[…] The effect of that whole drama […] had a severe effect on my mental health because then I thought ‘Now I am not a team player’ and ‘Now I am not good at my job’ and that ended up with me getting very anxious and depressed at one point and then having to go on medication from the doctors as a result of it*.*” (Digital forensics technician)*

#### Psychological support

Talking to a psychologist provided the participants with a safe haven where they could check in with their feelings and mental state and receive reassurance. Having access to a professional who was a specialist in the field, and seeing the same psychologist repeatedly helped to facilitate a level of trust. Six participants considered it important to receive regular psychological support or assessments as a preventive measure. Sessions with a psychologist were also beneficial even when participants were already beginning to feel the negative effects of their job. Recent changes in organisational procedures due to changes in funding led to a reduction in the frequency of assessment sessions and a change in the procedures required to access psychological support. These changes were seen to limit the support available and could possibly deter participants from requesting this support when needed. For example, in some units, the assessment procedure was switched from face to face to an online survey. In other units, participants that were previously able to directly contact the psychologist to request a session themselves, were now required to enter a lengthy process which could take several months before obtaining counselling.

*“I personally believe we should probably be having check-ins with a psychologist*, *at least once a year*, *because most mental health support requires you to identify you have a problem—which is hard—know where to get help for that problem*, *then take the brave and difficult step of speaking out and then going to get that help*. *Then that must be the right help for you*, *and you must not get discouraged if you need to go find different help*. *So*, *it is a lot of steps between you and getting the help you need*.*” (Digital forensics technician)*

*“We do have to see a psychotherapist every 6 months*. *Realistically it is probably not enough of an appointment*. *It used to be if you reached a point where you were struggling to cope you could make yourself a referral back to that psychotherapist for sessions that are more regular*. *This now has been stopped so you have a mandatory 6 months screening but if you ask for help and ask to see her*, *you will not*. *You are just referred to the occupational health queue which is a lengthy sort of queueing system for a phone appointment which will not really offer you any assistance and then it is months of waiting to get any psychotherapy or counselling*. *With the current climate we have had to just develop our own strategies to deal with things*.*” (Detective)*

#### Physical workplace

The actual physical environment in the workplace was important in helping the participants feel comfortable while doing their work. Having enough physical space, natural light, TVs and access to the gym were perceived as factors enhancing participants’ comfort and provided a connection with the outside world, whilst reducing feelings of isolation. However, opinions varied regarding the office layouts, one participant found that the noise occurring due to open-plan offices was disruptive, while another pointed out they would not like to be alone while viewing distressing material. Another difficulty of working in an open-plan office was that it increased the likelihood of accidentally viewing distressing material.

*“Where I work*, *I have a lot of space*. *I like space*, *it makes me feel relaxed*. *If I was confined*, *I think like everyone*, *you would feel a bit constricted*, *you cannot really move around*.” *(Digital forensics technician)*

*“The open plan*, *I do not think it is great*. *It has been an issue […] because there are indecent images everywhere*. *[…] Many practitioners minimize the material*, *so generally they do not put it on a big screen*. *I think it is small practical things like that which mitigate the risk*, *but I think it is still risky*.” *(Digital forensics technician)*

#### Volume and frequency of exposure

Participants described varying frequencies, durations, and types of exposure. This was often dictated by their role. Some participants reported having to look at distressing imagery on a daily basis, while others only tended to view it in weekly or monthly intervals. However, because of the uniqueness of each criminal case, the duration and frequency of exposure could vary widely, and participants were not always able to predict when, and for how long they would have to look at distressing material. Added to this variability were the instances when exposure happened accidentally, such as when discovering illegal material that is not initially relevant to the current case that is being worked on. Similarly, issues were also related to workplace aspects such as sharing computers and working in open-plan offices where participants often saw their colleagues’ work. This unexpected exposure sometimes led to responses of distress.

*“Outside my room they [the colleagues] were reviewing a paedophile’s computer and they had it on a massive screen*. *So normally […]*, *if they are reviewing something*, *they have it small*, *so it is only them that are really exposed to it*. *This officer did not really know that*, *so they had it on a big screen and they had it there all day and every time I came out of my office*, *I was seeing stuff on the screen*. *Most of it was regular pornography and regular abuse*, *nothing too extreme*, *well it is all extreme but nothing really bad and I said a couple of times to the member of staff that I was with that they do not need it that big*, *but they did not seem to do anything about it*.*” (Digital forensics technician)*

Participants reported that the issue of accidental exposure was tackled in some units by organising separate spaces where staff could analyse distressing material. One participant also mentioned the organisation was in the process of implementing daily limits, which restrict the length of time each employee had to spend viewing distressing material. Exposure to distressing material was also modulated by staff rotations, employees in specific roles being moved to a different unit after a certain amount of time. Although the purpose of rotations was to facilitate training, this process also modulated exposure to specific types of material, due to different types of cases that were being worked on in specific units. For example, in one unit the participants may only work on cases exclusively involving CSAM. Although in other units the participants would still be working these types of cases, they may also work on other types of cases, such as fraud and murder. Interestingly, while this difference in the rates of exposure to CSAM between units was mentioned by two digital forensic technicians, it was not acknowledged by one of the managers. A similar opinion of management in regard to exposure to child sexual abuse cases across units was also voiced by one of the participants who talked to management about this issue. Three participants perceived having a mix of cases or tasks may be less taxing on their emotional state than being frequently exposed to CSAM. In a similar vein, one participant suggested rotations across departments where employees would do another type of job and be exposed to less distressing material. However, as stated by one participant, some employees may be more resilient to this type of work, and therefore deliberately choose to work in the child sexual abuse investigations unit, thus not needing rotations. This was also voiced by one of the managers, who said that some of the employees doing rotations in the child sexual abuse investigations unit prefer to remain in that unit due to environmental factors. The role of environmental factors such as moving teams, the work location and the stress associated with these changes were also mentioned by another manager, being seen to impact on the employees’ willingness to work in certain units.

*“I highlighted [to management] that maybe some people were better suited to some things than others and I put forward the idea that maybe instead of a rotation everyone has to do you ask for volunteers [to go and work in the child exploitation unit]*. *It was suggested to me that no one will volunteer*. *However*, *I pointed out that people do apply for jobs in the child exploitation unit and so they must know that they are ok with it*. *I wonder if that in itself*, *maybe you volunteer there for one year and then the next volunteer goes in*. *You could argue that there are not enough volunteers to sustain that*, *but I think that would work*. *If I was asked ‘Would you like to go to the child exploitation unit or to some murder scenes*?*’ I would volunteer for the murder scenes because I would be ok with that*.*” (Digital forensics technician)*

*“You do indecent images [cases] on both [units] but at least you get the odd*, *it will sound really weird*, *but at least you get the odd murder or fraud which somehow feels gentler*, *which is like a palate cleanser*.*” (Digital forensics technician)*

*“I became very frustrated because they [management] could not differentiate between an environment where it [indecent images cases] is constantly around you all the time*, *when you cannot escape it unless you leave the office*, *versus an environment where you can get away from it and you might do two or three indecent image cases back to back but the next one might be a fraud or a murder or something else and you might have a good break in-between*. *I found it very frustrating that the management were unable to differentiate*.” *(Digital forensics technician)*

*“It could take you two months to get used to where you are working and then in a few months’ time you could be rotating again and that stays on your mind*. *‘Where will I be going in a few months*?*’*. *I do not know who I am going to get either*. *You build a good team and the strength of the team could vary quite a lot*. *I know who I can trust with something sensitive*, *who I think this might just break you if I give you this case*. *You cannot learn that with every rotation*.*” (Manager)*

*“When people come to work here [in the child exploitation unit] they are generally concerned about the work and its content*. *However*, *you find at the end of their rotation that the people who worked here want to stay*.” *(Manager)*

Exposure was also modulated by the gradual introduction within law enforcement of intelligent systems that can process evidence autonomously. The process through which this technology worked was by comparing the material found on a suspect’s device with images and videos stored in an official database containing pre-existing indecent imagery of children. The use of this technology was viewed by the participants as efficient in reducing the volume of material they needed to view. However, despite these positive improvements, frequency of exposure was still relatively high due to ever increasing workloads. While some of the cases could be sent to contractors, this was only possible for the cases that did not contain CSAM. This in turn increased the participants’ frequency of exposure to CSAM. Two participants also talked about the development and implementation of artificial intelligence tools that will be able to automatically categorise CSAM, further reducing their need to view this type of material. However, opinions were split regarding the perceived effectiveness of such tools, one participant feeling particularly positive about these tools’ high performance (over 90% accuracy) while another participant commented on the fact that they did not show perfect accuracy in performance.

*“The tools we use are moving us away from having to view them as much and we tend to focus more on the technical side of things*. *While we would still view them*, *it is sort of incidental to what we are doing in a way because what we have now are a lot of tools that can do the categorization of the images for us which is great because you do not have to weave through hundreds of thousands of images that you have seen time and time again*. *You can ignore these and just concentrate on the images that have not been done*. *It does take a lot of that away from us which is great*.” *(Digital forensics technician)*

*“We can only send out [to contractors] all the fraud or anything that is not indecent but anything sexual we have to do it ourselves which then you think* ‘*Actually I quite like to have the option of doing a fraud job rather than have to worry about this but if all you can do is illicit images of children*, *just extreme pornography*…*’*. *It would just be nice if they provide you with a break away from it to do something else*.*” (Digital forensics technician)*

*“They are now developing artificial intelligence categorisations which is amazing*! *One of the tools we were looking at was 92% correct on categorisation of an image or video which is fantastic*. *It is better than what we do haha*. *I think you know*, *anything we can do to minimise the distress*.” *(Digital forensics technician)*

*“There is new software coming which is looking at machine learning and trying to get computers to do it for you [e*.*g*. *classification of indecent images of children] but I do not think it will ever be 100% machines*. *There always has to be some human outside because machines get it wrong still quite a lot of the time*.” *(Manager)*

#### Rationale for doing the job

Participants discussed the importance of feeling that they are making a difference in the world by helping with the prosecution and conviction of offenders. Discovering that their investigation contributed to an appropriate sentence being given to perpetrators in court helped to give the participants meaning, closure and a justification for having viewed the material, particularly when working on live cases, where there is often an immediate outcome. Conversely, not having information about the case outcome or a sentencing that was perceived to be inappropriate to the offence committed was found to be demotivating.

*“Immediately when they go to court*, *if I did not go with them*, *they will come back and tell me what the result was and I think it is great reinforcement for what you do*, *definitely*!” *(Digital forensics technician)*

*“You work on something from 6 months ago and you might not hear back from it for a year or two…You might not hear back at all and so you feel like that although you know that is part of the job*, *you feel you are being subjected to these images but with no real understanding of what contribution you have had*.*” (Digital forensics technician)*

#### Social support

Having supportive colleagues provided opportunities to share feelings and experiences and offered companionship. This sharing then translated into the participants taking active steps in shielding each other from the distress that can be induced by the material. For example, participants would offer to swap cases that might be too stressful for another colleague due to their personal circumstances. Also, participants worked on developing their skills in order to improve coping. For example, one participant took a course in mental health in order to better support themselves and their colleagues.

*“What we tend to do is look out for each other […]*. *So*, *if we pick up a job that is going to be upsetting then we will swap jobs*, *or I will just say ‘Give me that job*’. *If my colleague gets a case and says*, *‘These children are the same age as mine’ I would grab the folder and quickly look and say*, *‘Do not worry about that*, *I will do this one’*. *Obviously you try to protect each other*.*” (Digital forensics technician)*

*“Because I do not get too stressed out about it then I think that maybe I would rather do it than someone who would get stressed out by it*.” *(Digital forensics technician)*

#### Resilience

An individual’s ability to cope with viewing distressing material was also considered to be an important factor. This related to their innate ability to be resilient, and appropriate staff selection procedures that may be able to assess individuals’ resilience to this specific type of work. Furthermore, this could help to establish their willingness to work with distressing material, and to develop organisational training methods intended to promote resilience. Resilience was not perceived to be an impenetrable force, but rather influenced by repeated exposure to distressing material and by current and old (e.g. history of abuse) events in the participants’ personal lives. Regarding resilience training, the participants noted that this type of training was not part of their preparation. However, participants were strongly in favour of receiving such training.

*“They should offer pre-training before you actually deal with indecent images as well*. *That is something they do not offer*. *You just dive in and do the job […]*. *There is training for what I do*, *for my job analysis side*, *but there is no training on how to view and manage the indecent images*. *I think that is something they could do*.*” (Digital forensics technician)*

*“There is a new trend emerging of officers who have never done this type of work and never wanted to do this type of work being drafted into this unit to do work that involves being immediately exposed to images*. *[*…*] I think it is a ticking bomb*.” *(Detective)*

*“People have*, *in my experience*, *become vulnerable at different times and places*. *When you become vulnerable because of your own circumstances that is not necessarily a good time to be looking at this*. *If someone has just been divorced for instance*, *they are vulnerable*. *If they have just had a death in the family*, *they are vulnerable*.” *(Manager)*

## Discussion

LEP need to view potentially disturbing material as part of their cases. However, there is limited research examining LEP’s perceptions and experiences of working with such material. This study attempts to address this gap in knowledge, by examining UK LEP’s experiences with viewing distressing evidence. We provide information about their reactions to viewing distressing material, the impact of exposure, factors modulating their coping in this job together with recommendations aimed to facilitate their well-being.

Depictions of violence, the victims’ displays of emotions, features personally relevant to LEP, norm violations and the medium of the material strongly modulated LEP’s affective responses. These factors were also reported by other members of LE exposed to distressing material [6–10, 14] and are corroborated by empirical research [36–45], suggesting the results are generalizable. In line with previous research in law enforcement [6–10, 14, 46], LEP’s initial reaction to this material was one of distress. This manifested in short-term emotional and physical responses of shock and disgust. Over time, viewing distressing material was associated with reports of STS symptoms such as intrusive images and thoughts, flashbacks and nightmares. Additionally, more general changes were also reported such as a developed overprotectiveness of children and paranoia. While a few LEP reported longer lasting effects of exposure to distressing material, some reported mild and transient symptoms, indicating wide variation in the severity of symptoms reported. Although in our study we did not aim to measure levels of STS, these results mirror PTSD research which found that most people who are exposed to a traumatic event will not go on to develop long term psychopathology and, in those who develop PTSD, around half of them will recover within a year without professional help [47–49].

Looking at reasons behind this variation in the severity of the symptoms reported, it is possible that at the time of assessment LEP experienced different levels of desensitisation when viewing distressing material. Assuming that desensitisation is curvilinear [50–52], it is likely members of LE who reported being more affected did not yet reach a functional level of desensitisation. However, the role of desensitisation, if any, in protecting LEP from developing STS symptoms is yet to be investigated. In the meantime, literature suggests a more linear relationship between the frequency [28] and duration [53] of exposure to distressing material, and its subsequent impact on LEP’s STS symptoms. Also, there is evidence that desensitisation impacts on a person’s general well-being, leading to greater tolerance [54] and enjoyment [55] of violence and impaired interpersonal relationships [56, 57]. A few of the LEP in our study showed concerns regarding these effects and worried that desensitisation will lead to changes in their “moral compass”. These fears were also discussed in Powell et al. [9] in relation to members of LE having developed an “un-empathetic and flippant attitude” because of having viewed distressing material.

At the agency level, efforts to reduce LEP’s exposure to distressing material and workload manifested through the introduction of technological tools aiding members of LE in their investigations. LEP used hash-based technology which automatically processed CSAM by comparing it to existing databases of previously identified CSAM. This technology is widely used in different units across the world [58]. In the UK, members of LE rely on their own personal database, the Child Abuse Image Database [59], in combination with access to larger international databases such as the Interpol Child Sexual Exploitation Image database [60]. Although in early stages, recently, UK law enforcement agencies started implementing tools using machine learning that can automatically identify and categorise CSAM according to the UK grading system [61]. To our knowledge, the UK is the first country to use such an advanced image analysis tool that is also tailored to classifying CSAM according to its legislation. In contrast, the other tools available on the market use more low-level techniques of identifying CSAM and are prone to high numbers of false alarms [62]. However, introducing these tools is unlikely to solve the problem of reducing LEP’s exposure to other types of distressing material, which may also induce trauma symptoms. To our knowledge, technology able to identify other types of distressing material is not currently used within law enforcement organisations.

Apart from technological tools, LEP’s exposure to this material was modulated by organisational policies. For example, in some units, separate spaces were created for analysing potentially distressing material. Also, members of LE had daily limits on the length of time they were required to examine distressing material. Furthermore, LEP engaged in compulsory rotations, a practice which has also been observed in law enforcement units in countries such as Canada and the USA [14]. While previous research [14] is vague on the specifics of these rotations, the LEP in our study were required to move across units. This procedure modulated LEP’s frequency of exposure to CSAM due to the focus of specific units on only working child sexual abuse cases, while other units worked a mix of cases. Although overexposure to CSAM can easily occur in the child sexual abuse investigations units, it is also likely to happen in other units due to organisational procedures developed in response to workload, which required members of LE to send non-child sexual abuse cases to contractors, leaving them frequently having to work child sexual abuse cases themselves. LEP perceived working a mix of cases to be less emotionally taxing than being overexposed to CSAM due to only working child sexual abuse cases. This opinion is in line with research showing that frequency of exposure to distressing material was directly linked to STS symptoms [28], pointing towards the potential mental health consequences prompted by these organisational procedures.

To shift their focus, LEP’s coping strategies when viewing distressing material included mental preparation, shutting off their emotions, positive reappraisal and denial. The use of dark humour and talking were among the other strategies used, although these were not necessarily uniquely tied to the moments when LEP viewed the material. While these strategies were previously mentioned by members of LE in other studies [6–10, 14, 46], they are not an indicator of effective coping, and may lead to different outcomes when it comes to LEP’s well-being. Positive reappraisal has previously been associated with fewer STS symptoms in members of LE [28], insurance claims workers [63] and trauma responders [64]. Similarly, shutting off one’s emotions, a form of emotional detachment, was shown to lead to reduced emotional responses to distressing material [65]. Also, talking about distressing experiences may ameliorate the negative effects of trauma [66]. LEP often disclosed being able to comfortably talk about their experiences with colleagues, with whom they shared similar experiences, given they could not share these experiences with family and friends. These opinions are in line with research which showed that talking about traumatic experiences with family, friends outside of work [67] or supervisors [68] does not have an impact on professionals’ PTSD symptoms. Rather, only moderate levels of such talk with co-workers was found to ameliorate the effects of trauma in LEP, this being mediated by the ease of talking about traumatic experiences [68]. In contrast, coping through denial [28] and dark humour [69] were associated with worse STS scores. The use of maladaptive coping strategies such as denial suggest a lack of knowledge in regard to the usefulness of different viewing strategies, which itself is possibly fuelled by the absence of resilience training in LEP’s curriculum. Interestingly, in our study denial was only mentioned by female members of LE, suggesting there may be a gender difference in the coping strategies used when viewing distressing material. However, general research within law enforcement and stress does not seem to indicate there is a gender difference in LEP’s engagement in maladaptive coping strategies [70, 71].

Looking at other factors safeguarding LEP’s well-being, having a sense of satisfaction about one’s job, and being able to detach from work while off duty were considered as protective factors for professionals exposed to trauma [72, 73]. Engaging in activities such as yoga and meditation is believed to have the capacity to encourage emotional self-awareness [74, 75], which may ameliorate STS symptoms [76]. Self-care approaches such as physical exercise and healthy nutrition are also seen as protective in the development and management of STS symptoms [77–82], although research suggests they do not contribute to mitigating these symptoms [28, 83]. This is possibly because these activities are not able to single-handedly combat STS but rather act by boosting the efficacy of other procedures [84]. LEP in this study and in previous studies [6, 8, 9, 14] talked about these factors in relation to their lives, indicating awareness of the role and impact they have on their resilience. Unfortunately, having this awareness does not necessarily equate with actual engagement in self-care activities [83].

Social support was another important factor in LEP’s well-being. This view is supported by research which found social support decreases STS symptoms in members of LE [27, 28] and is concurred by other LEP’s reports [6, 8, 9, 14] and by psychological models of resilience [72, 73]. For the LEP in this study, social support included the formal support offered by management and during therapy and informal peer support. Apart from relying on their colleagues when expressing emotions associated with viewing distressing material, LEP also supported each other in the form of swapping cases perceived to be personally distressing. At the organisational level, the need to receive tangible support through specific procedures to assist LEP was vital when reporting difficulty with viewing distressing material. This is especially important considering that self-reported difficulty shows a strong association with STS symptoms [27, 28]. Apart from tangible support, feeling supported and understood by their organisation was of great value to LEP. Interestingly, research indicates that perceived social support rather than the actual social support received plays a bigger role in relation to PTSD symptoms [85]. Within law enforcement, the level of perceived organisational support may potentially serve as a mitigating factor in the aftermath of trauma [86, 87]. LEP in our study displayed ambivalence about the support they were receiving from their organisations. Having management that possess an understanding of LEP’s struggles, and in particular an ability to express this understanding, created feelings of support in members of LE. In contrast, organisational changes limiting access to psychological support possibly contributed to LEP viewing their organisation as unsupportive.

A sense of feeling supported and understood was also important in the context of psychological interventions. While LEP in this study perceived psychological interventions as contributing to their well-being, a view in line with some other members of LE [6, 88], it is not an opinion universally held within the law enforcement literature. The reason behind these discrepancies may be due to differences in the perceived competency and understanding of the psychologists regarding the challenges and the nature of the material LEP view [14, 32]. Research points to the importance of making people feel understood during therapy, showing that perceived understanding during psychotherapy promotes clients’ satisfaction with therapy and increases treatment compliance [89, 90]. However, as a psychotherapy technique, making people feel deeply understood and supported (i.e. supportive counselling) seems to be less effective in reducing the symptoms associated with trauma, in comparison to Cognitive Behavioural Therapy [91]. Along the same lines, the use of exposure-based therapy and cognitive restructuring were found to lead to fewer symptoms of trauma in members of LE diagnosed with PTSD [92].

Discussions of social support are incomplete without considering the role of stigma. Police culture may discourage open expressions of vulnerability [32], admitting distress being seen by LEP as being able to potentially damage their reputation [93]. Stigma may thus stop members of LE from sharing their struggles, impacting on the support they receive. For example, stigma may influence LEP’s level of engagement with support services such as psychological interventions [32, 94]. Indeed, in a qualitative study, mental illness stigma was linked to decreased help-seeking and decreased resilience [95]. These issues were also voiced by LEP in our study, suggesting stigma may also have had an influence on their well-being.

### Practical recommendations

Our findings brought to attention two main areas of improvement that, if tackled, may better assist members of LE in working with distressing material. These appeal to improving personal resilience through training and to facilitating support in the workplace by addressing the organisation’s culture.

#### Training

LEP developed their coping strategies through trial and error, not having been offered any resilience training. As observed through LEP’s coping strategies, this absence of training may lead to the development of unhealthy coping strategies such as denial, alcohol consumption [28] etc. These maladaptive strategies may consequently have an impact on their personal well-being by affecting LEP’s mental health [28] and may also impact the organisation by influencing LEP’s turnover intentions [8]. Considering the potential impact of developing unhealthy coping strategies together with the registered changes in LEP’s access to support services, the need for supporting members of LE in developing effective coping strategies is strongly justified.

One potential area of training could be to focus on the development of cognitive reappraisal, a strategy which involves altering the way one thinks about a stimulus [96] in order to change its affective impact [97]. As illustrated by Fitzgerald et al. [98], this technique may have a protective effect in the development of PTSD symptoms. In their study, the researchers discovered that military veterans showing a deficiency in reappraisal also showed more overall PTSD symptoms. Similarly, in the context of viewing possibly traumatising material, positive reappraisal was found to be negatively associated with PTSD symptoms in adolescents exposed to media depicting a terrorist attack [99]. Along the same lines, cognitive reappraisal was associated with lesser total PTSD severity and greater positive affect [100], lower depression and lower state and trait anxiety [101], fewer subsequent trauma-related intrusions [102], and more effective down regulation of negative emotions [103].

Training may also focus on the development of mindfulness, a technique characterised by a focus on experiencing present-moment awareness of one’s thoughts, emotions and other experiences in the moment without attempts to suppress, judge or emotionally react to any stimuli [104, 105]. The role of mindfulness in mental well-being is illustrated by research showing that individuals with higher levels of mindfulness experience fewer PTSD and depression symptoms [106–109]. Mindfulness can be developed through mindfulness-based interventions such as Mindfulness Based Stress Reduction [105], Mindfulness Based Cognitive Therapy [110] etc. Among other things, these interventions have been associated with lowered negative affect [111, 112], less mind wandering [113–115] and increases in positive affect [111, 112]. Also, these techniques were linked to a reduction in the symptoms of depression and anxiety of both healthy [116–118] and people diagnosed with a mental health disorder [117, 119–122]. In the context of PTSD, individuals completing mindfulness-based interventions showed reductions in PTSD symptoms and an improvement in quality of life [123, 124].

LEP’s knowledge on the topic of resilience to trauma may also be enhanced through various materials. Resource materials such as ‘Trauma Stewardship: An Everyday Guide to Caring for Self While Caring for Others’ [125], ‘Help for the Helper: Self-Care Strategies for Managing Burnout and Stress’ [126] or ‘The Compassion Fatigue Workbook: Creative Tools for Transforming Compassion Fatigue and Vicarious Traumatization’ [127] may provide members of LE with information about self-care. Furthermore, LEP may access resources provided by programs such as the Supportive Heroes in Mental Health Foundational Training [128] who supply materials tailored to assist law enforcement professionals exposed to distressing material. Training should also focus on educating members of LE about how to identify and manage signs and symptoms of STS. This is relevant considering that LEP in our study described difficulty in self identifying symptoms of trauma. An example of such training is the “Certified Compassion Fatigue Specialist Training” [129] which was developed to improve an individual’s understanding and competency to identify and manage their own STS. The efficacy of this programme was proven on a sample of mental health professionals who completed this training and showed a significant decrease in STS scores [129].

#### Organisational culture

An organisation’s culture affects the way in which its employees think, make decisions and ultimately the way in which they perceive information, feel and act [130]. Thus, by addressing the organisation’s culture, we can tackle multiple aspects identified to play a role in LEP’s well-being. For example, creating a culture of wellness which normalizes mental health difficulties and improves mental health literacy may tackle mental health stigma and encourage members of LE to seek professional help [131]. Looking at the role of organisational culture in relation to interpersonal relationships in the workplace, promoting a culture of transformational leadership within teams encourages the development of trust among team members [132] and increases levels of team cohesion [133]. This is of relevance considering LEP’s reliance on their colleagues for support.

Organisational culture is also important when considering policies. As we have seen, LEP identified drawbacks and suggested improvements in a range of practices such as rotations, handing cases, support services and employee recruitment. While we have no information regarding the processes which led to decisions regarding these procedures, we feel it is important to emphasise that a culture promoting employee involvement in making decisions about organisational changes is essential for the successful implementation of change [134–136]. This is because including employees in the decision making process allows them to solve problems that matter to them within the organisation, builds participation, transparency, trust and commitment to the proposed changes [137].

### Directions for future research

Considering that in this study and in previous empirical studies [36–45] similar imagery features have been identified to lead to heightened emotional responses, a potential research avenue would be to investigate how these features could be manipulated with the scope of reducing LEP’s negative affect. Also, since LEP were at risk of being overexposed to distressing material, research should look at whether the efficacy of coping strategies and mitigating factors may be outweighed by a certain amount of exposure.

### Limitations

Although the LEP in this study worked in the same police agency, they had different roles and the time working in their current roles ranged from 1 year to more than 4 years. However, all LEP viewed distressing material as part of their jobs, making their experiences valuable to this study. Also, when it came to their direct experiences with viewing distressing material, these experiences were similar regardless of LEP’s role. Differences in experiences were only evidenced in relation to organisational procedures that varied depending on LEP’s job title. More specifically, only digital forensic technicians were required to engage in rotations, making this experience unique to them and to the managers who had new subordinates at every rotation. Given the possible stigma surrounding admitting any sign of weakness within police environments, LEP may have been selective in aspects of their experiences that they decided to disclose. The level of disclosure may also have been driven by the interviewer’s gender and age [138] and by the fact that the researcher was an “outsider” to the police world. However, generally LEP appeared to be comfortable and open during the interviews. Also, the interviewer’s gender may have in fact facilitated LEP’s disclosure of personal matters due to women being perceived to be more interested in one’s personal and emotional experiences [139].

### Conclusion

This paper articulates British LEP’s perceptions and experiences of working with distressing material from both a therapeutic and occupational safety perspective. LEP’s reported impact of viewing distressing material, the features of the material found to elicit strong affective responses, their coping strategies and the personal factors identified to mitigate the effects of viewing distressing material were similar to those reported in previous studies of law enforcement [6, 8–10, 14, 32] uniting them in their experiences. However, since these experiences were also modelled by LEP’s agencies, the unique contribution of this study is to provide insight into the impact of specific organisational procedures on LEP’s experiences. On one hand the organisation actively worked towards reducing LEP’s exposure to distressing material by investing in the introduction of new technological tools to assist members of LE in their investigations, creating separate spaces for viewing evidence and limiting the length of time LEP were allowed to view such evidence. On the other hand, they increased LEP’s exposure to distressing content through organisational procedures such as rotations and sending non-child sexual abuse cases to contractors and altered LEP’s access to support services. These procedures were driven by factors such as workload and funding, revealing the complex and contentious nature associated with making decisions regarding these aspects. Nevertheless, these policies were significant in their potential impact on LEP’s mental health. We hope the findings and recommendations which emerged from this study will further assist LEP’s well-being.

## Supporting information

S1 Table Participant characteristics. (DOCX)

S2 Table Interview questions. (DOCX)

## List of abbreviations

CSAM: child sexual abuse material
LEP: law enforcement professionals
LE: law enforcement
STS: secondary traumatic stress
PTSD: post-traumatic stress disorder
UK: United Kingdom
USA: United States of America

## References

[1] ACPO. The ACPO good practice guide for managers of e-Crime investigation. 2011 October [Cited 2020 May 6]. In: Digital Detective [Internet]. Available from: https://www.digital-detective.net/digital-forensics-documents/ACPO_Good_Practice_and_Advice_for_Manager_of_e-Crime-Investigation.pdf

[2] Aiken M, Mc Mahon C, Haughton C, O’Neill L, O’Carroll E. A consideration of the social impact of cybercrime: examples from hacking, piracy, and child abuse material online. Contemp Soc Sci. 2016;11: 373–391.

[3] IWF. 2019 Annual Report. 2020 Apr 27 [Cited 2020 May 11]. In: Internet Watch Foundation [Internet]. Available from: https://www.iwf.org.uk/report/iwf-2019-annual-report-zero-tolerance

[4] IWF. 2018 Annual Report. 2019 Apr 25 [Cited 2020 May 11]. In: Internet Watch Foundation [Internet]. Available from: https://www.iwf.org.uk/report/2018-annual-report

[5] HO News Team. Fact Sheet on online Child Sexual Exploitation and Abuse. 2019 Jun 25 [Cited 2020 Jun 3]. Available from: https://homeofficemedia.blog.gov.uk/2019/06/25/fact-sheet-on-online-child-sexual-exploitation-and-abuse/

[6] Burns CM, Morley J, Bradshaw R, Domene J. The Emotional Impact on and Coping Strategies Employed by Police Teams Investigating Internet Child Exploitation. Traumatol. 2008;14: 20–31.

[7] Krause M. Identifying and managing stress in child pornography and child exploitation investigators. J Police Crim Psychol. 2009;24: 22–29.

[8] Perez LM, Jones J, Englert DR, Sachau D. Secondary traumatic stress and burnout among law enforcement investigators exposed to disturbing media images. J Police Crim Psychol. 2010;25: 113–124.

[9] Powell M, Cassematis P, Benson M, Smallbone S, Wortley R. Police officers’ perceptions of their reactions to viewing internet child exploitation material. J Police Crim Psychol. 2015;30: 103–111.

[10] Stevenson J. Welfare considerations for supervisors managing child sexual abuse online units. M.Sc. Thesis, Middlesex University. 2007. Available from: https://www.shiftwellness.org/wp-content/uploads/2016/05/Bound-Bramshill-Study-2007.pdf

[11] Quick D, Choo K-KR. Impacts of increasing volume of digital forensic data: A survey and future research challenges. Digit Investig. 2014;11: 273–294.

[12] Irvine J. The darker side of computer forensics. 2011 July 18 [Cited 2020 May 10]. In: Forensic Focus [Internet]. Available from: https://www.forensicfocus.com/articles/the-darker-side-of-computer-forensics/

[13] Edelmann RJ. Exposure to child abuse images as part of one’s work: possible psychological implications. J Forens Psychiatry Psychol. 2010;21: 481–489.

[14] Fortune N, Rooney B, Kirwan GH. Supporting Law Enforcement Personnel Working with Distressing Material Online. Cyberpsychol Behav Soc Netw. 2018;21: 138–143. 10.1089/cyber.2016.0715 29048939

[15] Figley CR. Compassion fatigue: Toward a new understanding of the costs of caring. In: Stamm BH, editor. Secondary traumatic stress: Self-care issues for clinicians, researchers, and educators. Baltimore, MD, US: The Sidran Press, xxiii; 1995 pp. 3–28.

[16] American Psychiatric Association. Diagnostic and Statistical Manual of Mental Disorders (DSM-5®). American Psychiatric Pub; 2013 5 22.

[17] Cornille TA, Meyers TW. Secondary Traumatic Stress Among Child Protective Service Workers: Prevalence, Severity and Predictive Factors. Traumatol. 1999;5: 15–31.

[18] Johnson WB, Bertschinger M, Snell AK, Wilson A. Secondary trauma and ethical obligations for military psychologists: preserving compassion and competence in the crucible of combat. Psychol Serv. 2014;11: 68–74. 10.1037/a0033913 24564444

[19] Temitope KM, Williams M. Secondary traumatic stress, burnout and the role of resilience in New Zealand counsellors. New Zealand Journal of Counselling. 2015;35: 1–21.

[20] Bell S, Hopkin G, Forrester A. Exposure to Traumatic Events and the Experience of Burnout, Compassion Fatigue and Compassion Satisfaction among Prison Mental Health Staff: An Exploratory Survey. Issues Ment Health Nurs. 2019;40: 304–309. 10.1080/01612840.2018.1534911 30742547

[21] Perron BE, Hiltz BS. Burnout and secondary trauma among forensic interviewers of abused children. Child Adolesc Social Work J. 2006;23: 216–234.

[22] MacEachern AD, Dennis AA, Jackson S, Jindal-Snape D. Secondary Traumatic Stress: Prevalence and Symptomology Amongst Detective Officers Investigating Child Protection Cases. J. Police Crim Psychol. 2019;34: 165–174.

[23] Sollie H, Kop N, Euwema MC. Mental Resilience of Crime Scene Investigators: How Police Officers Perceive and Cope With the Impact of Demanding Work Situations. Crim Justice Behav. 2017;44: 1580–1603.

[24] Greinacher A, Derezza-Greeven C, Herzog W, Nikendei C. Secondary traumatization in first responders: a systematic review. Eur J Psychotraumatol. 2019;10: 1562840 10.1080/20008198.2018.1562840 30719236 PMC6346705

[25] Shakespeare-Finch J. Primary and secondary trauma in emergency personnel. Traumatol. 2011;17: 1.

[26] Cohen R, Leykin D, Golan-Hadari D, Lahad M. Exposure to traumatic events at work, posttraumatic symptoms and professional quality of life among midwives. Midwifery. 2017;50: 1–8. 10.1016/j.midw.2017.03.009 28347853

[27] Bourke ML, Craun SW. Secondary traumatic stress among Internet Crimes Against Children task force personnel: impact, risk factors, and coping strategies. Sex Abuse. 2014;26: 586–609. 10.1177/1079063213509411 24259539

[28] Bourke ML, Craun SW. Coping with secondary traumatic stress: Differences between UK and US child exploitation personnel. Traumatology: An International Journal. 2014;20: 57.

[29] Bride BE, Robinson MM, Yegidis B, Figley CR. Development and Validation of the Secondary Traumatic Stress Scale. Res Soc Work Pract. 2004;14: 27–35.

[30] Seigfried-Spellar KC. Assessing the Psychological Well-being and Coping Mechanisms of Law Enforcement Investigators vs. Digital Forensic Examiners of Child Pornography Investigations. J Police Crim Psychol. 2018;33: 215–226.

[31] Holt TJ, Blevins KR, Burruss GW. Examining the stress, satisfaction, and experiences of computer crime examiners. J Crime Justice. 2012;35: 35–52.

[32] Powell MB, Cassematis P, Benson MS, Smallbone S, Wortley R. Police officers’ perceptions of the challenges involved in Internet Child Exploitation investigation. Policing. 2014;37: 543–557.

[33] Mazzola JJ, Schonfeld IS, Spector PE. What qualitative research has taught us about occupational stress. Stress Health. 2011;27: 93–110. 10.1002/smi.1386 27486613

[34] InqScribe. Version 2.2.4 [software]. 2018 Feb [Cited 2020 Jun 20]. Available from: https://www.inqscribe.com/

[35] Braun V, Clarke V. Using thematic analysis in psychology. Qual Res Psychol. 2006;3: 77–101.

[36] Baumgartner T, Lutz K, Schmidt CF, Jäncke L. The emotional power of music: how music enhances the feeling of affective pictures. Brain Res. 2006;1075: 151–164. 10.1016/j.brainres.2005.12.065 16458860

[37] Ekman P, Levenson RW, Friesen WV. Autonomic nervous system activity distinguishes among emotions. Science. 1983;221: 1208–1210. 10.1126/science.6612338 6612338

[38] Ellard KK, Farchione TJ, Barlow DH. Relative effectiveness of emotion induction procedures and the role of personal relevance in a clinical sample: A comparison of film, images, and music. J Psychopathol Behav Assess. 2012;34: 232–243. 10.1007/s10862-011-9271-4 PMC8489569 34611377

[39] Hess U, Blairy S. Facial mimicry and emotional contagion to dynamic emotional facial expressions and their influence on decoding accuracy. Int J Psychophysiol. 2001;40: 129–141. 10.1016/s0167-8760(00)00161-6 11165351

[40] Magnée MJCM, Stekelenburg JJ, Kemner C, de Gelder B. Similar facial electromyographic responses to faces, voices, and body expressions. Neuroreport. 2007;18: 369–372. 10.1097/WNR.0b013e32801776e6 17435605

[41] Moody EJ, Reed CL, Van Bommel T, App B, McIntosh DN. Emotional Mimicry Beyond the Face?: Rapid Face and Body Responses to Facial Expressions. Soc Psychol Personal Sci. 2018;9: 844–852.

[42] Ousdal OT, Reckless GE, Server A, Andreassen OA, Jensen J. Effect of relevance on amygdala activation and association with the ventral striatum. Neuroimage. 2012;62: 95–101. 10.1016/j.neuroimage.2012.04.035 22546319

[43] Park J-Y, Gu B-M, Kang D-H, Shin Y-W, Choi C-H, Lee J-M, et al Integration of cross-modal emotional information in the human brain: an fMRI study. Cortex. 2010;46: 161–169. 10.1016/j.cortex.2008.06.008 18691703

[44] Sander D, Grafman J, Zalla T. The human amygdala: an evolved system for relevance detection. Rev Neurosci. 2003;14: 303–316. 10.1515/revneuro.2003.14.4.303 14640318

[45] Strathearn L, Kim S. Mothers’ amygdala response to positive or negative infant affect is modulated by personal relevance. Front Neurosci. 2013;7: 176 10.3389/fnins.2013.00176 24115918 PMC3792358

[46] MacEachern AD, Jindal-Snape D, Jackson S. Child Abuse Investigation: Police Officers and Secondary Traumatic Stress. Int J Occup Saf Ergon. 2011;17: 329–339. 10.1080/10803548.2011.11076898 22152500

[47] Fletcher S, Creamer M, Forbes D. Preventing post-traumatic stress disorder: are drugs the answer? Aust N Z J Psychiatry. 2010;44: 1064–1071. 10.3109/00048674.2010.509858 21070102

[48] Norris FH, Slone LB. The epidemiology of trauma and PTSD. In: Handbook of PTSD: Science and practice. Guildford Press; 2007 pp. 78–98.

[49] Ruzek JI, Brymer MJ, Jacobs AK, Layne CM, Vernberg EM, Watson PJ. Psychological first aid. J Ment Health Couns. 2007;29: 17–49.

[50] Mrug S, Madan A, Cook EW 3rd, Wright RA. Emotional and physiological desensitization to real-life and movie violence. J Youth Adolesc. 2015;44: 1092–1108. 10.1007/s10964-014-0202-z 25326900 PMC4393354

[51] Gaylord-Harden NK, Cunningham JA, Zelencik B. Effects of exposure to community violence on internalizing symptoms: does desensitization to violence occur in African American youth? J Abnorm Child Psychol. 2011;39: 711–719. 10.1007/s10802-011-9510-x 21505848

[52] Ng-Mak DS, Salzinger S, Feldman RS, Stueve CA. Pathologic adaptation to community violence among inner-city youth. Am J Orthopsychiatry. 2004;74: 196–208. 10.1037/0002-9432.74.2.196 15113248

[53] Feinstein A, Audet B, Waknine E. Witnessing images of extreme violence: a psychological study of journalists in the newsroom. JRSM Open. 2014;5: 205427041453332 10.1177/2054270414533323 25289144 PMC4100239

[54] Fanti KA, Vanman E, Henrich CC, Avraamides MN. Desensitization to media violence over a short period of time. Aggress Behav. 2009;35: 179–187. 10.1002/ab.20295 19172659

[55] Krahé B, Möller I, Huesmann LR, Kirwil L, Felber J, Berger A. Desensitization to media violence: links with habitual media violence exposure, aggressive cognitions, and aggressive behavior. J Pers Soc Psychol. 2011;100: 630–646. 10.1037/a0021711 21186935 PMC4522002

[56] Carnagey NL, Anderson CA, Bushman BJ. The effect of video game violence on physiological desensitization to real-life violence. J Exp Soc Psychol. 2007;43: 489–496.

[57] Bushman BJ, Anderson CA. Comfortably numb: desensitizing effects of violent media on helping others. Psychol Sci. 2009;20: 273–277. 10.1111/j.1467-9280.2009.02287.x 19207695

[58] ECPAT International. Trends in online child sexual abuse material. ECPAT International. 2018 Apr [Cited 2020 Apr 12]. In ECPAT [Internet]. Available from: https://www.ecpat.org/wp-content/uploads/2018/07/ECPAT-International-Report-Trends-in-Online-Child-Sexual-Abuse-Material-2018.pdf

[59] Home office. Child Abuse Image Database. In: The Government Digital Service [Internet]. 2018 May [cited 2020 Jul 8]. Available from: https://assets.publishing.service.gov.uk/government/uploads/system/uploads/attachment_data/file/759328/CAID_Brochure_May_2018_for_gov_uk.pdf

[60] Interpol. International Child Sexual Exploitation database. In: Interpol [Internet]. 2020 [Cited 2020 Jul 13]. Available from: https://www.interpol.int/en/Crimes/Crimes-against-children/International-Child-Sexual-Exploitation-database

[61] Home Office. Pioneering new tools to be rolled out in fight against child abusers. 2019 [Cited 2020 Jun 3]. Available from: https://www.gov.uk/government/news/pioneering-new-tools-to-be-rolled-out-in-fight-against-child-abusers

[62] Vitorino P, Avila S, Perez M, Rocha A. Leveraging deep neural networks to fight child pornography in the age of social media. J Vis Commun Image Represent. 2018;50: 303–313.

[63] Ludick M. Analyses of experiences of vicarious traumatisation in short-term insurance claims workers. Doctoral Thesis, University of Witwaresrand. 2013. Available from: https://core.ac.uk/download/pdf/39671844.pdf

[64] Burnett HJ Jr, Wahl K. The compassion fatigue and resilience connection: A survey of resilience, compassion fatigue, burnout, and compassion satisfaction among trauma responders. Int J Emerg Ment Health. 2015;17: 318–326.

[65] Walter H, von Kalckreuth A, Schardt D, Stephan A, Goschke T, Erk S. The temporal dynamics of voluntary emotion regulation. PLoS One. 2009;4: e6726 10.1371/journal.pone.0006726 21949675 PMC3175755

[66] Bootzin RR. Examining the Theory and Clinical Utility of Writing About Emotional Experiences. Psychol Sci. 1997;8: 167–169.

[67] Southwick SM, Morgan CA 3rd, Rosenberg R. Social sharing of Gulf War experiences: association with trauma-related psychological symptoms. J Nerv Ment Dis. 2000;188: 695–700. 10.1097/00005053-200010000-00008 11048819

[68] Stephens C, Long N. Communication with police supervisors and peers as a buffer of work-related traumatic stress. J Organ Behav. 2000;21: 407–424.

[69] Craun SW, Bourke ML. The use of humor to cope with secondary traumatic stress. J Child Sex Abus. 2014;23: 840–852. 10.1080/10538712.2014.949395 25085244

[70] He N, Zhao J, Archbold CA. Gender and police stress: The convergent and divergent impact of work environment, work‐family conflict, and stress coping mechanisms of female and male police officers. Policing: An International Journal of Police Strategies & Management. 2002;25: 687–708.

[71] Ortega A, Brenner S-O, Leather P. Occupational Stress, Coping and Personality in the Police: An SEM Study. International Journal of Police Science & Management. 2007;9: 36–50.

[72] Kapoulitsas M, Corcoran T. Compassion fatigue and resilience: A qualitative analysis of social work practice. Qual Soc Work. 2015;14: 86–101.

[73] Ludick M, Figley CR. Toward a mechanism for secondary trauma induction and reduction: Reimagining a theory of secondary traumatic stress. Traumatol. 2017;23: 112–123.

[74] Dale LP, Mattison AM, Greening K, Galen G, Neace WP, Matacin ML. Yoga Workshop Impacts Psychological Functioning and Mood of Women With Self-Reported History of Eating Disorders. Eat Disord. 2009;17: 422–434.

[75] Nielsen L, Kaszniak AW. Awareness of subtle emotional feelings: a comparison of long-term meditators and nonmeditators. Emotion. 2006;6: 392–405. 10.1037/1528-3542.6.3.392 16938081

[76] Killian KD. Helping Till It Hurts? A Multimethod Study of Compassion Fatigue, Burnout, and Self-Care in Clinicians Working With Trauma Survivors. Traumatol. 2008;14: 32–44.

[77] Coetzee SK, Klopper HC. Compassion fatigue within nursing practice: a concept analysis. Nurs Health Sci. 2010;12: 235–243. 10.1111/j.1442-2018.2010.00526.x 20602697

[78] Dane B. Child welfare workers: An innovative approach for interacting with secondary trauma. J Soc Work Educ. 2000;36: 27–38.

[79] Hesse AR. Secondary trauma: How working with trauma survivors affects therapists. Clin Soc Work J. 2002;30: 293–309.

[80] Iliffe G, Steed LG. Exploring the Counselor’s Experience of Working With Perpetrators and Survivors of Domestic Violence. J Interpers Violence. 2000;15: 393–412.

[81] Neville K, Cole DA. The Relationships Among Health Promotion Behaviors, Compassion Fatigue, Burnout, and Compassion Satisfaction in Nurses Practicing in Community Medical Center. JONA: The Journal of Nursing Administration. 2013;43: 348. 10.1097/NNA.0b013e3182942c23 23708503

[82] Zadeh S, Gamba N, Hudson C, Wiener L. Taking care of care providers: a wellness program for pediatric nurses. J Pediatr Oncol Nurs. 2012;29: 294–299. 10.1177/1043454212451793 22907685 PMC5178976

[83] Bober T, Regehr C. Strategies for Reducing Secondary or Vicarious Trauma: Do They Work? Brief Treat Crisis Interv. 2006;6: 1–9.

[84] Powers MB, Medina JL, Burns S, Kauffman BY, Monfils M, Asmundson GJG, et al Exercise Augmentation of Exposure Therapy for PTSD: Rationale and Pilot Efficacy Data. Cogn Behav Ther. 2015;44: 314–327. 10.1080/16506073.2015.1012740 25706090 PMC4464974

[85] Prati G, Pietrantoni L. The relation of perceived and received social support to mental health among first responders: a meta-analytic review. J Community Psychol. 2010;38: 403–417.

[86] Maguen S, Metzler TJ, McCaslin SE, Inslicht SS, Henn-Haase C, Neylan TC, et al Routine work environment stress and PTSD symptoms in police officers. J Nerv Ment Dis. 2009;197: 754–760. 10.1097/NMD.0b013e3181b975f8 19829204 PMC3974929

[87] Regehr C, Johanis D, Dimitropoulos G, Bartram C, Hope G. The Police Officer and the Public Inquiry: A Qualitative Inquiry into the Aftermath of Workplace Trauma. Brief Treat Crisis Interv. 2003;3(4).

[88] Tucker JM. Police officer willingness to use stress intervention services: The role of perceived organizational support (POS), confidentiality and stigma. International Journal of Emergency Mental Health and Human Resilience. 2015;17: 304.

[89] Ackerman SJ, Hilsenroth MJ. A review of therapist characteristics and techniques positively impacting the therapeutic alliance. Clin Psychol Rev. 2003;23: 1–33. 10.1016/s0272-7358(02)00146-0 12559992

[90] Reis HT, Clark MS, Pereira Gray DJ, Tsai F-F, Brown JB, Stewart M, et al Measuring Responsiveness in the Therapeutic Relationship: A Patient Perspective. Basic Appl Soc Psych. 2008;30: 339–348.

[91] Forneris CA, Gartlehner G, Brownley KA, Gaynes BN, Sonis J, Coker-Schwimmer E, et al Interventions to prevent post-traumatic stress disorder: a systematic review. Am J Prev Med. 2013;44: 635–650. 10.1016/j.amepre.2013.02.013 23683982

[92] Peres JFP, Foerster B, Santana LG, Fereira MD, Nasello AG, Savoia M, et al Police officers under attack: resilience implications of an fMRI study. J Psychiatr Res. 2011;45: 727–734. 10.1016/j.jpsychires.2010.11.004 21159352

[93] Fair DJ. Counseling cops: learning how to navigate the law enforcement subculture. Annals of the American Psychotherapy Association. 2009;12(4): 50–52.

[94] Wright R, Powell MB, Ridge D. Child abuse investigation. Policing: an international journal of police strategies & management. 2006;29: 498–512.

[95] Crowe A, Averett P, Glass JS. Mental illness stigma, psychological resilience, and help seeking: What are the relationships? Ment Health Prev. 2016;4: 63–68.

[96] Gross JJ. The Emerging Field of Emotion Regulation: An Integrative Review. Rev Gen Psychol. 1998;2: 271–299.

[97] Cutuli D. Cognitive reappraisal and expressive suppression strategies role in the emotion regulation: an overview on their modulatory effects and neural correlates. Front Syst Neurosci. 2014;8: 175 10.3389/fnsys.2014.00175 25285072 PMC4168764

[98] Fitzgerald JM, Gorka SM, Kujawa A, DiGangi JA, Proescher E, Greenstein JE, et al Neural indices of emotional reactivity and regulation predict course of PTSD symptoms in combat-exposed veterans. Prog Neuropsychopharmacol Biol Psychiatry. 2018;82: 255–262. 10.1016/j.pnpbp.2017.11.005 29122638

[99] Jenness JL, Jager-Hyman S, Heleniak C, Beck AT, Sheridan MA, McLaughlin KA. Catastrophizing, rumination, and reappraisal prospectively predict adolescent PTSD symptom onset following a terrorist attack. Depress Anxiety. 2016;33: 1039–1047. 10.1002/da.22548 27557454 PMC5325818

[100] Boden MT, Bonn-Miller MO, Kashdan TB, Alvarez J, Gross JJ. The interactive effects of emotional clarity and cognitive reappraisal in Posttraumatic Stress Disorder. J Anxiety Disord. 2012;26: 233–238. 10.1016/j.janxdis.2011.11.007 22169054

[101] Moore SA, Zoellner LA, Mollenholt N. Are expressive suppression and cognitive reappraisal associated with stress-related symptoms? Behav Res Ther. 2008;46: 993–1000. 10.1016/j.brat.2008.05.001 18687419 PMC2629793

[102] Nickerson A, Garber B, Liddell BJ, Litz BT, Hofmann SG, Asnaani A, et al Impact of Cognitive Reappraisal on Negative Affect, Heart Rate, and Intrusive Memories in Traumatized Refugees. Clin Psychol Sci. 2017;5: 497–512.

[103] Butler O, Willmund G, Gleich T, Zimmermann P, Lindenberger U, Gallinat J, et al Cognitive Reappraisal and Expressive Suppression of Negative Emotion in Combat-Related Posttraumatic Stress Disorder: A Functional MRI Study. Cognit Ther Res. 2019;43: 236–246.

[104] Bishop SR, Lau M, Shapiro S, Carlson L, Anderson ND, Carmody J, et al Mindfulness: A Proposed Operational Definition. Clinical Psychology: Science and Practice. 2006 pp. 230–241.

[105] Kabat-Zinn J. Using the Wisdom of Your Body and Mind to Face Stress, Pain, and Illness. New York, NY: Bantam Doubleday Dell; 1990

[106] Barr N, Davis JP, Diguiseppi G, Keeling M, Castro C. Direct and indirect effects of mindfulness, PTSD, and depression on self-stigma of mental illness in OEF/OIF veterans. Psychol Trauma. 2019. 10.1037/tra0000535 31804109

[107] Barr N, Keeling M, Castro C. Associations between mindfulness, PTSD, and depression in combat deployed post-9/11 military veterans. Mindfulness; 2019.

[108] Im S, Follette VM. Rumination and mindfulness related to multiple types of trauma exposure. Transl Issues Psychol Sci. 2016;2: 395–407.

[109] Huang Q, Zhang Q, An Y, Xu W. The relationship between dispositional mindfulness and PTSD/PTG among firefighters: The mediating role of emotion regulation. Pers Individ Dif. 2019;151: 109492.

[110] Morgan D. Mindfulness-based cognitive therapy for depression. A new approach to preventing relapse. Psychotherapy Research. 2003;13: 123–125. 10.1080/713869628 22475168

[111] Brockman R, Ciarrochi J, Parker P, Kashdan T. Emotion regulation strategies in daily life: mindfulness, cognitive reappraisal and emotion suppression. Cogn Behav Ther. 2017;46: 91–113. 10.1080/16506073.2016.1218926 27684649

[112] Jha AP, Stanley EA, Kiyonaga A, Wong L, Gelfand L. Examining the protective effects of mindfulness training on working memory capacity and affective experience. Emotion. 2010;10: 54–64. 10.1037/a0018438 20141302

[113] Morrison AB, Goolsarran M, Rogers SL, Jha AP. Taming a wandering attention: short-form mindfulness training in student cohorts. Front Hum Neurosci. 2014;7: 897 10.3389/fnhum.2013.00897 24431994 PMC3880932

[114] Mrazek MD, Smallwood J, Schooler JW. Mindfulness and mind-wandering: finding convergence through opposing constructs. Emotion. 2012;12: 442–448. 10.1037/a0026678 22309719

[115] Mrazek MD, Franklin MS, Phillips DT, Baird B, Schooler JW. Mindfulness training improves working memory capacity and GRE performance while reducing mind wandering. Psychol Sci. 2013;24: 776–781. 10.1177/0956797612459659 23538911

[116] Chiesa A, Serretti A. Mindfulness-based stress reduction for stress management in healthy people: a review and meta-analysis. J Altern Complement Med. 2009;15: 593–600. 10.1089/acm.2008.0495 19432513

[117] Fjorback LO, Arendt M, Ørnbøl E, Fink P, Walach H. Mindfulness-Based Stress Reduction and Mindfulness-Based Cognitive Therapy—a systematic review of randomized controlled trials. Acta Psychiatr Scand. 2011;124: 102–119. 10.1111/j.1600-0447.2011.01704.x 21534932

[118] Khoury B, Sharma M, Rush SE, Fournier C. Mindfulness-based stress reduction for healthy individuals: A meta-analysis. J Psychosom Res. 2015;78: 519–528. 10.1016/j.jpsychores.2015.03.009 25818837

[119] McCarney RW, Schulz J, Grey AR. Effectiveness of mindfulness-based therapies in reducing symptoms of depression: A meta-analysis. Eur J Psychother Couns. 2012;14: 279–299.

[120] Piet J, Hougaard E. The effect of mindfulness-based cognitive therapy for prevention of relapse in recurrent major depressive disorder: a systematic review and meta-analysis. Clin Psychol Rev. 2011;31: 1032–1040. 10.1016/j.cpr.2011.05.002 21802618

[121] Strauss C, Cavanagh K, Oliver A, Pettman D. Mindfulness-based interventions for people diagnosed with a current episode of an anxiety or depressive disorder: a meta-analysis of randomised controlled trials. PLoS One. 2014;9: e96110 10.1371/journal.pone.0096110 24763812 PMC3999148

[122] Vøllestad J, Nielsen MB, Nielsen GH. Mindfulness-and acceptance-based interventions for anxiety disorders: A systematic review and meta-analysis. Br J Clin Psychol. 2012;51: 239–260. 10.1111/j.2044-8260.2011.02024.x 22803933

[123] Polusny MA, Erbes CR, Thuras P, Moran A, Lamberty GJ, Collins RC, et al Mindfulness-Based Stress Reduction for Posttraumatic Stress Disorder Among Veterans: A Randomized Clinical Trial. JAMA. 2015;314: 456–465. 10.1001/jama.2015.8361 26241597

[124] Jasbi M, Sadeghi Bahmani D, Karami G, Omidbeygi M, Peyravi M, Panahi A, et al Influence of adjuvant mindfulness-based cognitive therapy (MBCT) on symptoms of post-traumatic stress disorder (PTSD) in veterans—results from a randomized control study. Cogn Behav Ther. 2018;47: 431–446. 10.1080/16506073.2018.1445773 29893182

[125] van Dernoot Lipsky L., Burk C. Trauma Stewardship: An Everyday Guide to Caring for Self While Caring for Others. California: Berrett-Koehler Publishers; 2009.

[126] Rothschild B. Help for the helper: The psychophysiology of compassion fatigue and vicarious trauma. New York, NY, US: WW Norton & Co; 2006.

[127] Mathieu F. The compassion fatigue workbook: Creative tools for transforming compassion fatigue and vicarious traumatization. New York, NY: Routledge; 2012.

[128] shiftwellness.org [Internet]. Supporting Heroes in Mental Health Foundational Training [cited 20 Oct 2020]. Available from: https://www.shiftwellness.org/

[129] Gentry JE, Baggerly J, Baranowsky A. Training-as-treatment: effectiveness of the Certified Compassion Fatigue Specialist Training. Int J Emerg Ment Health. 2004;6: 147–155. 15481476

[130] Schein EH. Organizational culture. Am Psychol. 1990;45: 109–119.

[131] Carlan PE, Nored LS. An examination of officer stress: should police departments implement mandatory counseling? J Police Crim Psychol. 2008;23: 8–15.

[132] Arnold KA, Barling J, Kevin KE. Transformational leadership or the iron cage: which predicts trust, commitment and team efficacy? Leadership & Organization Development Journal. 2001;22: 315–320.

[133] Sparks JR, Schenk JA. Explaining the effects of transformational leadership: an investigation of the effects of higher-order motives in multilevel marketing organizations. J Organ Behav. 2001;22: 849–869.

[134] Lofquist EA. Doomed to Fail: A Case Study of Change Implementation Collapse In the Norwegian Civil Aviation Industry. Journal of Change Management. 2011;11: 223–243.

[135] Haffar M, Al-Karaghouli W, Ghoneim A. The mediating effect of individual readiness for change in the relationship between organisational culture and TQM implementation. Total Qual Manage Bus Excel. 2013;24: 693–706.

[136] Haffar M, Al-Karaghouli W, Ghoneim A. An empirical investigation of the influence of organizational culture on individual readiness for change in Syrian manufacturing organizations. Journal of Organizational Change Management. 2014;27: 5–22.

[137] Edwards K, Prætorius T, Nielsen AP. A Model of Cascading Change: Orchestrating Planned and Emergent Change to Ensure Employee Participation. Journal of Change Management. 2020; 1–27.

[138] Richards H, Emslie C. The “doctor” or the “girl from the University”? Considering the influence of professional roles on qualitative interviewing. Fam Pract. 2000;17: 71–75. 10.1093/fampra/17.1.71 10673494

[139] Lohan M. Extending feminist methodologies: Researching masculinities and technologies. Re) searching Women Dublin: IPA 2000; 167–187.

