# Correction: Understanding and supporting law enforcement professionals working with distressing material: Findings from a qualitative study

**DOI:** 10.1371/journal.pone.0253682

**Published:** 2021-06-17

**Authors:** Cristina-Bianca Denk-Florea, Benjamin Gancz, Amalia Gomoiu, Martin Ingram, Reuben Moreton, Frank Pollick

The following grant number is missing from the Funding statement: ES/P000681/1. The correct Funding statement is as follows: This study was carried out with PhD funding received by CDF from the Economic and Social Research Council (ESRC) under the grant reference numbers ES/R5009381/1 and ES/P000681/1.
