# Supplementary material for: Understanding and supporting law enforcement professionals working with distressing material: Findings from a qualitative study
Source: PLoS One. 2020 Nov 25;15(11):e0242808. doi: 10.1371/journal.pone.0242808 (PMC7688122; doi:10.1371/journal.pone.0242808)
Supplement: S1 Table — (DOCX) [file pone.0242808.s001.docx]

**S1 Table.** **Participant characteristics.**

| **Characteristic** | | **Number (%)** | **Gender distribution** |
| --- | --- | --- | --- |
| Gender | Female | 6 (27%) | - |
|  | Male | 16 (73%) | - |
| Ethnicity | White | 16 (72%) | Female = 4; Male = 12 |
|  | Asian | 2 (9%) | Female = 2; Male = 0 |
|  | Mixed | 1 (5%) | Female = 0; Male = 1 |
| Education | O level/GCSE equivalent | 1 (5%) | Female = 0; Male = 1 |
|  | A level or equivalent | 4 (18%) | Female = 1; Male = 3 |
|  | HNC/HND/GNVQ | 1 (5%) | Female = 0; Male = 1 |
|  | Undergraduate degree | 6 (27%) | Female = 3; Male = 3 |
|  | Postgraduate degree | 7 (32%) | Female = 2; Male = 5 |
| Age at time of interview | 21-30 | 6 (27%) | Female = 3; Male = 3 |
|  | 31-40 | 5 (23%) | Female = 3; Male = 2 |
|  | 41-50 | 6 (27%) | Female = 0; Male = 6 |
|  | 51-60 | 1 (5%) | Female = 0; Male = 1 |
|  | 61-70 | 2 (9%) | Female = 0; Male = 2 |
| Job role | Digital forensics technician | 13 (59%) | Female = 4; Male = 9 |
|  | Manager | 4 (18%) | Female = 1; Male = 3 |
|  | Investigator | 3 (13%) | Female = 1; Male = 2 |
|  | Supervisor | 1 (5%) | Female = 0; Male = 1 |
|  | Detective | 1 (5%) | Female = 0; Male = 1 |
| Years in current post | 1-2 years | 5 (22%) | Female = 3; Male = 2 |
|  | 2-3 years | 3 (14%) | Female = 1; Male = 2 |
|  | 3-4 years | 1 (5%) | Female = 0; Male = 1 |
|  | 4+ years | 11 (50%) | Female = 2; Male = 9 |
| Marital status | Single | 3 (13%) | Female = 2; Male = 1 |
|  | Civil partnered | 3 (13%) | Female = 1; Male = 2 |
|  | Married | 6 (27%) | Female = 3; Male = 3 |
|  | Divorced | 1 (5%) | Female = 0; Male = 1 |
| Dependent* | Had dependent | 8 (36%) | Female = 2; Male = 6 |
|  | Did not have dependent | 8 (36%) | Female = 4; Male = 4 |

**Note**: Numbers do not always add to 22 participants due to participants not providing this information. *Dependents = babies and children.
