# Supplementary material for: Understanding and supporting law enforcement professionals working with distressing material: Findings from a qualitative study
Source: PLoS One. 2020 Nov 25;15(11):e0242808. doi: 10.1371/journal.pone.0242808 (PMC7688122; doi:10.1371/journal.pone.0242808)
Supplement: S2 Table — (DOCX) [file pone.0242808.s002.docx]

**S2 Table. Interview questions**

| 1. What is your current role? |
| --- |
| 2. Can you describe the nature of your work? |
| 3. Are you or have you ever been required to examine images or videos through your role? |
| 4. If yes, what type of material have you been exposed to or are you normally exposed to? |
| 5. What is your subjective experience of working with this material? i.e. its affect, if at all, on you personally. |
| 6. What in the images or video materials made you react in that way? Can you identify any specific themes and/or features of the material which evoked the reactions you told me about? |
| 7. Is there anything that you believe helps you cope with viewing material? |
| 8. Are there any aspects related to your general work at the agency that you feel may affect or help you with analysing the material? |
